# Editorial: Physical education, schools and lifelong habits

**DOI:** 10.3389/fspor.2023.1180587

**Published:** 2023-04-25

**Authors:** Javier Cachón-Zagalaz, Víctor Arufe-Giraldez, Ángel Ramón Romance-García, María Luisa Zagalaz-Sánchez

**Affiliations:** ^1^ Departamento de Didáctica de la Expresión Musical, Plástica y Corporal, Facultad de Humanidades y Ciencias de la Educación, University of Jaén, Jaén, Spain; ^2^ Departamento de Didácticas Específicas y Métodos de Investigación y Diagnóstico en Educación, Facultad de Educación, University of A Coruña, A Coruña, Spain; ^3^ Departamento de Didáctica de las Lenguas, las Artes y el Deporte, Facultad de Ciencias de la Educación, University of Málaga, Málaga, Spain

**Keywords:** editorial, physical education, school, lifelong habits, teacher

**Editorial on the Research Topic** Physical education, schools and lifelong habits

The philosophy of Frontiers journals is to publish innovative topics, stimulate collaboration and accelerate science. This is what we have achieved with this Research Topic dedicated to Physical Education, Schools and Lifestyle. Although only four articles have been published, the expertise of their authors, the variety of their origins and the similarity of their themes, which revolve around Education and Health, has meant that just a few days before the closure of this issue, 1,000 people have read them, which endorses their scientific and academic interest.

To emphasize their importance, we have included in this editorial a micro-summary of the four articles published in chronological order:

1. Lewis et al.'s “*Nothing Is Gonna Change If We Don't Care for Everyone”: A Narrative Inquiry* is an investigation on urban indigenous youth in an after-school physical activity wellness program. Is written by 4 Canadian authors from educational and kinesiological backgrounds who implement the Growing Young Movers (GYM) after-school program based on physical activity, which reports good results for youth in the North Central neighborhood of Regina, Saskatchewan.
2. *Study protocol of “From Science 2 School”—prevalence of sports and physical exercise linked to omnivorous, vegetarian and vegan, diets among Austrian secondary schools* by Wirnitzer et al. is written by 9 researchers from the education and sports science departments of the Universities of Innsbruck, Stands and Linz. Its goal is to overcome the lack of knowledge about physical activity, sports, and exercise coupled with alternative diets (such as vegetarianism and veganism). They conclude that, with a sustainable healthy lifestyle, a healthy transition from childhood to adulthood occurs, which can lead to healthier generations in the long-term.
3. Sollerhed's original research article is *Perceived insufficient pedagogical content knowledge in teaching movement and physical activity. Experiences from an action-oriented study among educators in early childhood education and care.* Its author is a professor at the Faculty of Teacher Training, Kristianstad University (Sweden). This research studies the behavior and preparation of teachers to teach movement and physical activity (MoPA) as fundamental activities for the health and development of children. It concludes that educators working in Early Childhood Education and Care (ECEC) need to improve pedagogical content knowledge for adequate teaching in MoPA, which is important for children's current development and future health.
4. Finally there is Zhang et al.'s *Design, methodology, and baseline of Eastern China Student Health and Wellbeing Cohort Study*. The authors consist of 13 researchers from 7 Chinese universities and fields of education and medical knowledge. They present a program whose objective is to focus on the development of specific diseases of students (myopia, obesity, high blood pressure and mental health), with a longitudinal study planned until 2035.

In summary, these 4 articles deal with physical activity and health. They originate from universities as far apart as Canada, Sweden, Austria and China, three of them studying students and one studying teachers. Likewise, the authors of this editorial also come from the field of physical activity and health and we teach and do research in different and distant Spanish universities (A Coruña, Málaga and Jaén). We are interested in this content because we believe that Physical Education is an important part of child and youth development that will help maintain quality of life and better health in the future.

